# Backbone spiking sequence as a basis for preplay, replay, and default states in human cortex

**DOI:** 10.1038/s41467-023-40440-5

**Published:** 2023-08-07

**Authors:** Alex P. Vaz, John H. Wittig, Sara K. Inati, Kareem A. Zaghloul

**Affiliations:** 1 Department of Neurosurgery, University of Pennsylvania, Philadelphia, PA 19104 USA; 2 Surgical Neurology Branch, NINDS, National Institutes of Health, Bethesda, MD 20892 USA; 3 Office of the Clinical Director, NINDS, National Institutes of Health, Bethesda, MD 20892 USA

**Keywords:** Hippocampus, Long-term memory

## Abstract

Sequences of spiking activity have been heavily implicated as potential substrates of memory formation and retrieval across many species. A parallel line of recent evidence also asserts that sequential activity may arise from and be constrained by pre-existing network structure. Here we reconcile these two lines of research in the human brain by measuring single unit spiking sequences in the temporal lobe cortex as participants perform an episodic memory task. We find the presence of an average backbone spiking sequence identified during pre-task rest that is stable over time and different cognitive states. We further demonstrate that these backbone sequences are composed of both rigid and flexible sequence elements, and that flexible elements within these sequences serve to promote memory specificity when forming and retrieving new memories. These results support the hypothesis that pre-existing network dynamics serve as a scaffold for ongoing neural activity in the human cortex.

## Abstract (web summary)

Sequential neural spiking activity is a potential substrate for learning and memory across species. Here, the authors showed spiking in the human cortex forms an average backbone sequence, and flexibility around this backbone is associated with cognition.

## Introduction

Sequences of spiking activity are commonly observed in populations of neurons and are implicated as potential building blocks of information^1^. Consequently, spiking sequences have garnered much interest as potential neural substrates for learning and memory^2,3^. Hippocampal spiking sequences emerge during behavioral experience and are later replayed during periods of rest^4–9^. Replay of spiking sequences that are relevant for memory retrieval has also been described in the cortex of rodents^9–11^ and humans^12^. The evidence that the replay of spiking sequences is specific to individual memories has suggested that individual sequences may emerge to represent different experiences.

**Fig. 1:**
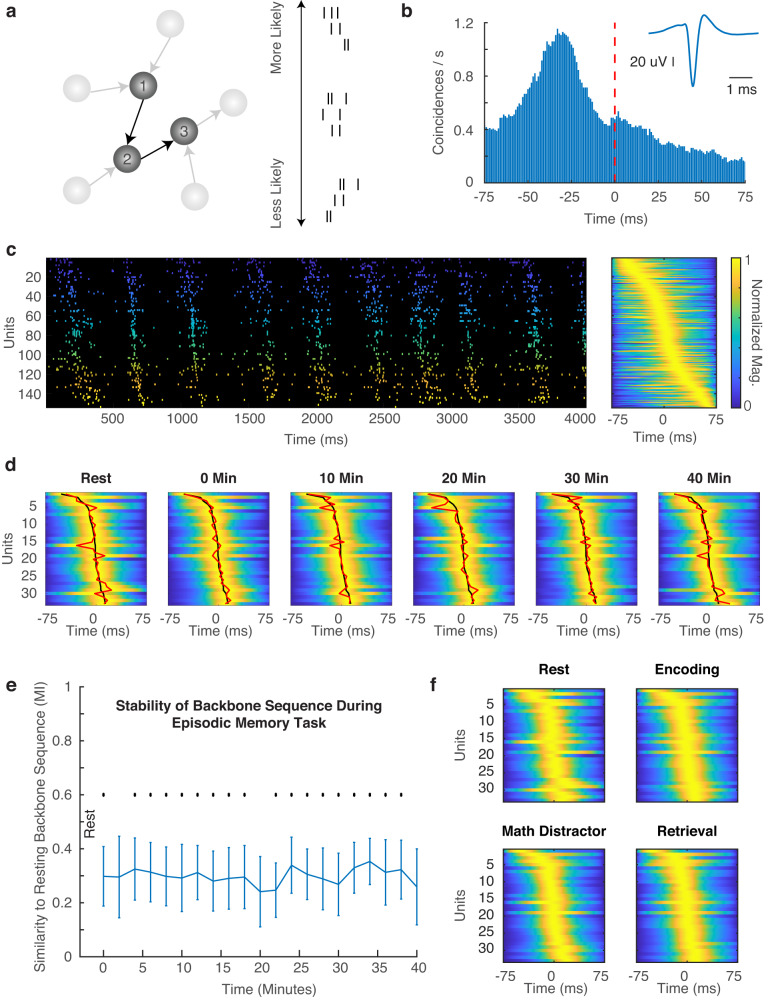
Human cortical backbone sequences persist across time and cognitive states. **a** Conceptual schematic demonstrating how a backbone sequence may arise from a given network simply by the pre-existing synaptic connections between neurons. **b** Cross-correlation of the spike train of a single unit with the summed spike trains of all other units from a given epoch. This unit had a peak firing time of ~30 ms before the other units in the population. The average waveform of the unit is shown in the inset. **c** A representative spike raster (left) demonstrating the ordering given by the corresponding backbone sequence (right). Cooler and hotter colors in the raster indicate units that, respectively, fire earlier and later in the backbone sequence. Colors in the backbone sequence are normalized between 0 and 1 for ease of visualization, and the associated color bar is shown on the right. **d** Backbone sequences extracted from 2 min non-overlapping epochs from rest to 40 min into the behavioral task. Red lines indicate the bin-specific backbone sequence while black lines indicate the backbone sequence extracted from the whole session. Here we show a separate patient than in (**c**) with fewer units in order to allow for visualization of changes to the backbone sequence over each epoch. **e** The backbone sequence across participants was consistent across the behavioral task regardless of the cognitive state at any point in time. Sequence similarity over time is calculated here as the similarity between the resting backbone sequence and the backbone sequence extracted from each subsequent 2 min non-overlapping bin. Error bars represent SEM across participants. Significance of each bin is denoted as a black dot (*N* = 6 participants, one-sided t-test against a null of 0), and testing for multiple comparisons is corrected by FDR (*q* = 0.05). **f** Average backbone sequences calculated for each different cognitive state during the episodic memory task. Sequences are shown ordered according to the whole session backbone sequence.

A parallel line of evidence, however, has suggested that sequential neural activity may arise simply from the pre-existing structure of the circuits of interest^13^. Spontaneous hippocampal spiking activity is organized into sequences^14,15^, and these sequences are present in early development^16^ and even guided by the birthdate of individual neurons^17^. These observations have led to the suggestion that sequential neural activity is intrinsically hard wired, resulting in a backbone sequence of neural activity that a given population is predisposed towards regardless of cognitive state^1,13,18^ (Fig. 1a). In this framework, recent experience is then not ascribed a completely new pattern of sequential spiking activity, but is constrained and influenced by the pre-existing underlying network dynamics^19,20^. Recent evidence supports this claim, with both sensory responses and memory representations being constrained by network activity present at baseline^21–23^.

The potential for a backbone sequence implies that the brain may select from a pre-existing repertoire of activity for use with later cognition. This phenomenon has been explicitly termed preplay and has been robustly demonstrated in rodents^24–26^. The ability to embed new memories into pre-existing neural activity also implies the existence of rigid and flexible elements of a putative backbone sequence. Loose adherence to the backbone sequence may be a consequence of rigid elements that are constrained by the underlying network connectivity, while variability around the backbone sequence may be a consequence of flexible elements that allow for the creation of novel and specific memory representations^27–29^. Empirical and theoretical studies have suggested that rigid and flexible elements are subserved by strongly connected fast-firing neurons and weakly connected slow-firing neurons, respectively^18^.

Spiking sequences have recently been described in the human cortex^12^, but it remains unknown if stereotypical backbone sequences exist in human cortex that can be reliably measured across different cognitive states. Here, we examine population spiking activity captured from the human anterior temporal lobe and find the presence of backbone sequences that remain relatively consistent over the course of an entire experimental session in which participants perform a verbal episodic memory task. The backbone sequence persists through rest, memory formation and retrieval, and distractor blocks of the task. Backbone sequences observed during the rest period are similar to those used later during memory formation, suggesting that preplay is also a feature of human cortical spiking activity. We distinguish rigid and flexible components of the backbone sequences and demonstrate that flexible elements are essential for the specificity of individual memories. Taken together, our results provide evidence for backbone sequences in the human cortex, and more generally for the existence of predetermined network dynamics in the human brain.

## Results

To investigate the possibility that backbone sequences of spiking activity existing in the human cortex, we collected single unit spiking activity from the anterior temporal lobe in in 6 participants (2 female; 34.8 ± 4.7 years; mean ± SEM) using a microelectrode array (MEA) implanted in each participant^12,30,31^. We adopted a previously reported methodology to calculate the average spike time latencies between each neuron and the summed population activity from all other units in each temporal epoch of interest^21,32^ (see Methods). The time of the peak cross-correlogram for each unit defines the temporal index of that unit as compared to the population spiking activity (Fig. 1b). After repeating this process for all units, we ordered all units by their respective temporal indices to generate an overall backbone sequence that describes the average sequential ordering of population activity over a specified length of time (Fig. 1c). We found a significant relation between the normalized ranks of individual units and the spatial distance between them, suggesting that physical distance between units can help shape the average sequential firing of the population (Fig. S1).

We extracted backbone sequences at different intervals over the course of a 40-min recording session as participants performed a verbal paired associates episodic memory task (see “Methods”). We divided each experimental session into 2-min non-overlapping epochs, including a 2-min rest period before the task had started. In a representative participant, there is remarkable similarity between the rest sequence and the subsequent backbone sequences extracted from each epoch (Fig. 1d). This consistency is significant across all participants across the 40 min of recording time, even though the patients were alternating between encoding periods, math distractors, and retrieval periods (Fig. 1e; FDR corrected for multiple comparisons). We also examined how similar the backbone sequence in each 2-min epoch is to all other epochs and found that the similarity between all epochs is also significantly greater than expected by chance across participants (Fig. S2). Surrogate distributions created by shuffling the timing of spiking activity or the identity of individual units do not exhibit such similarity between epochs (Fig. S3). Sequence similarity between epochs increases with the duration of the temporal epoch used to extract each backbone sequence (Fig. S4). Moreover, to investigate whether sequence stability was driven by the earlier or latter half of each sequence, we divided the backbone sequence of each epoch into two halves and recalculated sequence similarity over time. Across participants, we found no significant difference in sequence similarity between the early and late halves of the backbone sequences (MI difference = 0.030 ± 0.029; paired t-test, t(5) = 1.03, p > 0.05).

To confirm that the backbone sequences are consistent across these different cognitive states, we aggregated all data separately from the rest, encoding, math distractors, and retrieval periods of the task and computed a separate average backbone sequence for each state (Fig. 1f). We quantified the similarity between backbone sequences extracted from each pair of cognitive states in every session. Across participants, the resulting average sequence similarity across all pairs is significantly higher than chance (average MI = 0.42 ± 0.13; t(5) = 3.24, p = 0.023), and no pair of cognitive states exhibit a similarity significantly different than any other (1-way ANOVA, F(5) = 0.24, p > 0.05).

**Fig. 2:**
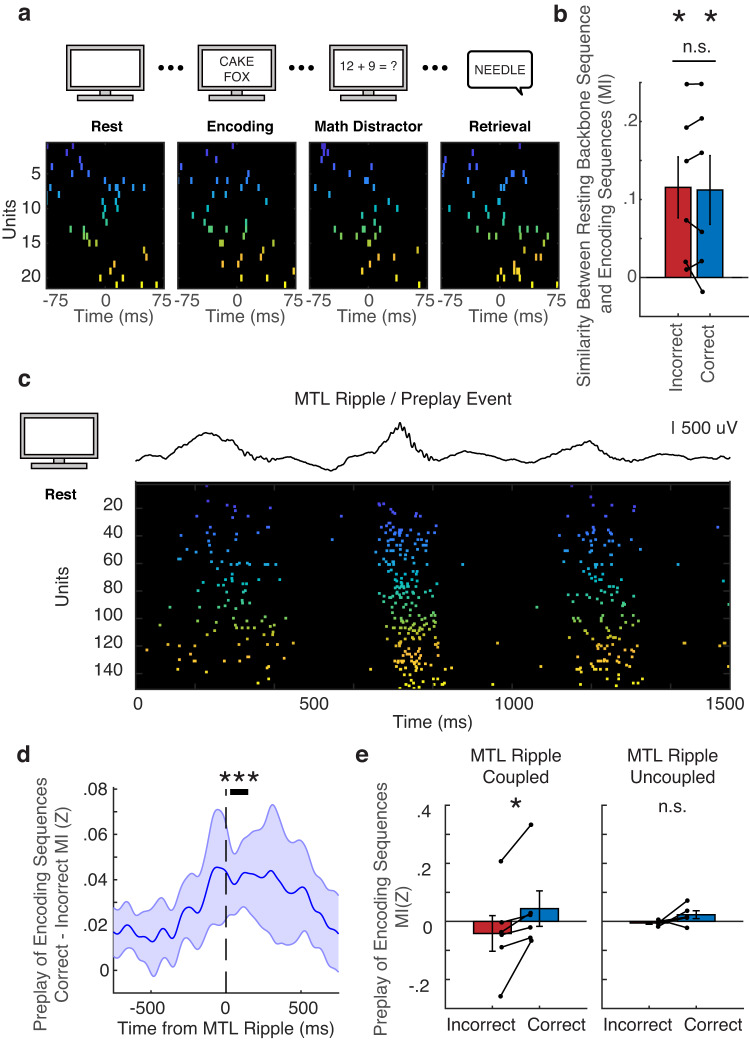
Preplay of memory-relevant spiking sequences. **a** Four individual spiking sequences from different cognitive periods in a testing session that are all significantly similar to one another. Each sequence shown is separated from the previous sequence by ~10 min in order to span the entire recording session. **b** Comparison of resting backbone sequence (2 min before task began) to spiking sequences during subsequent memory encoding epochs (one-sided t-test against 0 as null, incorrect: t(5) = 2.95, *p* = 0.0159; correct: t(5) = 2.54, *p* = 0.0259). (*) indicates *p* < 0.05 and n.s. indicates not significant. Error bars indicate SEM across participants. **c** Representative example of an MTL ripple co-occurring with a significant preplay sequence during the rest period. **d** Average similarity between resting cortical sequences and encoding sequences triggered to the onset of MTL ripples. Significant difference in similarity between correct versus incorrect trials is indicated by the bar (cluster-based permutation procedure correcting for multiple comparisons, *p* <0.001), indicating that MTL ripples precede cortical preplay by ~150 ms. Error bars indicate SEM across participants. **e** Sequence similarity between cortical spiking sequences during rest stratified based on if they were coupled to an MTL ripple (left) or not (right). Only MTL coupled preplay events demonstrate a significant difference in similarity between subsequently correctly and incorrectly encoded memories (N-way ANOVA with post hoc Tukey’s test, *p* = 0.034). Error bars indicate SEM across participants. *N* = 6 participants for all statistical tests in this figure.

We were interested in whether individual bursts of spiking activity that emerge during episodic memory formation exhibit a sequential order that is similar to these backbone sequences. Previous reports have suggested that individual sequences of spiking activity used during cognition may be selected from a pre-existing repertoire, a phenomenon referred to as preplay^24^. We implemented a previously reported spiking burst detection method to extract individual sequences based on the population spike rate exceeding a given threshold^12^ (see “Methods”). Burst events extracted in this manner are typically on the order of 100 ms (for correct encoding and retrieval, 108.0 ± 10.4 ms and 107.8 ± 10.8 ms, respectively). We subsequently found compelling examples of single sequences during rest that appear similar to sequences occurring in later parts of the episodic memory task (Fig. 2a). To quantify this similarity, we compared the backbone sequence extracted using the 2-min rest epoch to individual sequences observed as participants encoded word pair associations into memory. Individual sequences during both correct and incorrect encoding trials are significantly similar to the resting epoch backbone sequence across participants (Fig. 2b; one-sided t-test against 0 as null, incorrect: t(5) = 2.95, *p* = 0.0159; correct: t(5) = 2.54, *p* = 0.0259). We similarly examined the sequences present immediately before vocalization during memory retrieval (Fig. S5). The extent to which encoding and retrieval sequences are similar to the resting epoch backbone sequence is not different between correct and incorrect trials, suggesting that the sequence of spiking activity observed in a population of cortical neurons is relatively constrained regardless of the final cognitive outcome.

Although the individual sequences that emerge during memory encoding are similar to the average backbone sequence observed during rest, we were motivated to understand if any particular resting state sequence may be preferentially used later in successful memory encoding. Based on previous evidence that preplay of spiking sequences may be related to ripple oscillations in the rodent hippocampus^24^ and that cortical sequence replay in humans is coupled to MTL ripples^12^, we hypothesized that cortical spiking sequences that were coupled in time to MTL ripples may be preferentially used as patterns during later correct memory encoding. We found several examples of significant preplay events co-occurring with MTL ripples during the rest period (Fig. 2c). The spiking activity during these preplay events is in turn locked to the troughs of ripple oscillations recorded in the cortical electrodes (Fig. S6). We therefore quantified the temporal relationship between ripples observed in MTL electrodes and the preplay of spiking sequences observed in later memory encoding trials (Fig. 2d). In the ~150 ms following the onset of MTL ripples, we found that resting spiking sequences are on average significantly more similar to subsequent correct encoding trials than incorrect trials (*p* < 0.001, permutation-based cluster procedure, see “Methods”). We therefore classified any cortical sequence during rest as an MTL ripple coupled event if it fell within 150 ms of the start of an MTL ripple. We found that coupled sequences are on average significantly more similar to individual sequences observed during correct encoding trials compared to incorrect trials (Fig. 2e left; N-way ANOVA with post hoc Tukey’s test, *p* = 0.034). We found that this difference is not significant when examining uncoupled sequences during the rest period (Fig. 2e right).

**Fig. 3:**
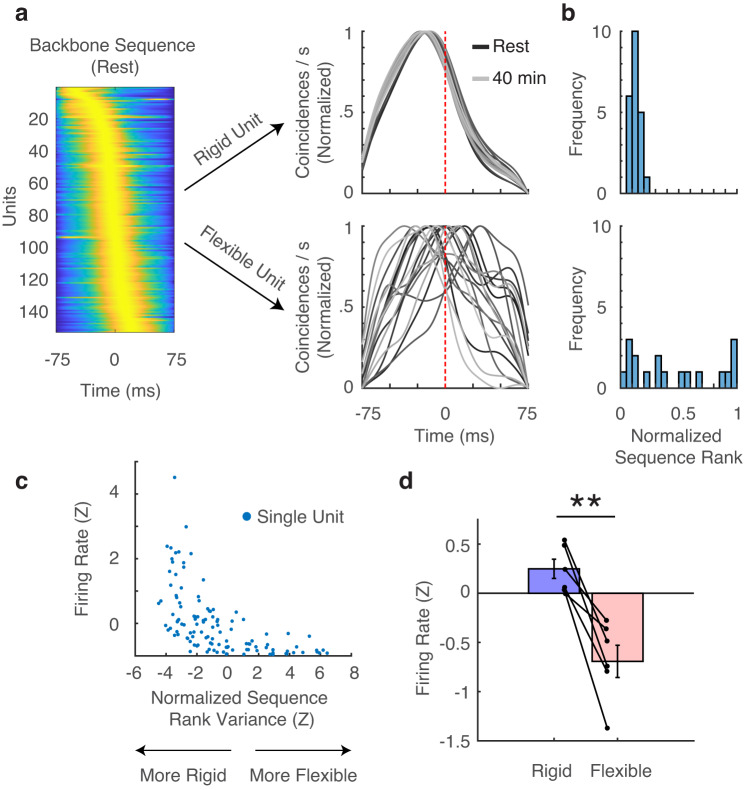
Backbone sequences have rigid and flexible elements. **a** Representative backbone sequence from a given participant (left) and two separate units are shown with individual traces indicating the cross-correlations of the spike train of that unit with the summed spike trains of all other units in the population (right). The rigid unit has a highly consistent temporal spiking relationship with the population activity whereas the flexible unit is much more inconsistent. Darker and lighter lines indicate earlier and later in the behavioral session, respectively. **b** Corresponding histograms of the normalized sequence rank for each 2 min bin for each unit across the entire recording session. **c** Relationship between Z-scored normalized sequence rank variance and firing rate for all units from a representative session. More negative values along the x-axis indicate a higher degree of rigidity. Each blue dot represents one isolated unit from this session. **d** Rigid units demonstrated significantly higher firing rates than flexible units across all participants (*N* = 6 participants, two-sided t-test, t(5) = 5.58, *p* = 0.003). (**) indicates *p* < 0.01. Error bars indicate SEM across participants.

Our data suggest that the sequential order of spiking activity in a population of cortical neurons remains relatively consistent during cognition. However, in order to account for the possibility that different individual sequences may be used to represent different memories, we hypothesized that backbone sequences should not be uniformly rigid over time but rather should be composed of both rigid and flexible sequence elements^27^. In a backbone sequence from a representative participant (Fig. 3a, left), we indeed found units whose temporal relationship to the population activity is very stable while other units have a temporal position that varies substantially across different backbone sequences extracted from different temporal epochs (Fig. 3a, right). We quantified the amount of variation by assessing the variance of the normalized rank of each unit across backbone sequences calculated from all 2-min epochs across the entire recording session (Fig. 3b). We compared the variance of each unit’s normalized rank position to a distribution of empirical variances obtained by chance via a shuffling procedure to generate a z-scored value that is comparable across participants (see “Methods”). We therefore classified a unit as rigid or flexible if the z-score in question was <−1 or >1, respectively, yielding 68.2 ± 11.3% rigid units and 16.0 ± 5.5% flexible units across participants (mean ± SEM). As expected, the time varying spike rates of rigid units are more highly correlated with one another than for flexible units (rigid: *r* = 0.141 ± 0.053; flexible: *r* = 0.030 ± 0.016; rigid to flexible: *r* = 0.048 ± 0.023).

Given this classification, an immediately testable hypothesis based on both empirical and theoretical studies is that rigid and flexible units should have high and low firing rates, respectively^18^. In a representative session, the firing rate is clearly negatively correlated with the flexibility of the neuron within a backbone sequence (Fig. 3c). This relationship is robust across all participants (Fig. 3d; paired t-test, t(5) = 5.58, *p* = 0.003). The relation between unit classification and firing rate raises the possibility that rigid and flexible units may reflect inhibitory and excitatory neurons, respectively^18^. We therefore examined whether we could distinguish rigid and flexible units based on waveform properties. We measured the valley-to-peak and half-peak-width for all units^33^, but found no significant difference between rigid and flexible units (Fig. S7). We were also interested in whether rigid and flexible units preferentially occupy the beginning or ends of each backbone sequence. We plotted the fraction of observed rigid units per normalized sequence rank for all participants against a null distribution where unit identities were randomly assigned. However, we found no significant difference between the observed and chance, indicating that rigid and flexible units in our data are likely uniformly distributed throughout the sequence (Fig. S8). Moreover, both types of units exhibit significant increases in spike rate ~50 ms from the onset of MTL ripples (Fig. S9).

While these results are consistent with previous reports, we were concerned that the underlying spike rates of each unit may in fact bias the classification into either being flexible or rigid. We therefore performed a control analysis in which we downsampled the spike rate of each rigid unit to match the spike rates of flexible units within each participant. Even when underlying spike rates between the two sets of units are matched in this manner, rigid units maintain their rigidity (Fig. S10). We also performed a second control analysis in which we modeled each spike train as an inhomogeneous Poisson process, therefore randomizing spike times while maintaining the overall spike counts. In this case, we observed a uniform decrease in the number of rigid units suggesting that the rigidity of these units is not simply due to their higher firing rates (Fig. S11). We reproduced all our main findings using both more conservative and more liberal thresholds for declaring a unit as rigid or flexible (Fig. S12). We additionally reproduced these results by using an alternative metric of unit rigidity within a sequence in which we classified each unit based on the change in average sequence similarity to the resting backbone sequence after randomly shuffling the sequence position of each unit (Fig. S13, see “Methods”).

**Fig. 4:**
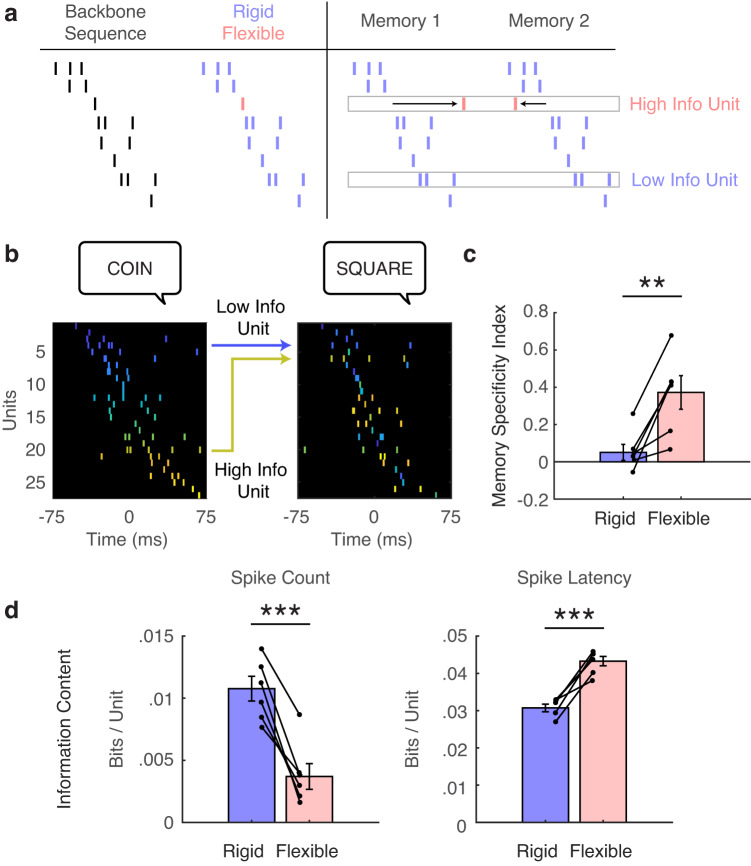
Flexible elements underlie memory specificity of spiking sequences. **a** Conceptual diagram depicting how backbone sequences are composed of rigid and flexible elements (left) and how flexible elements may rearrange to form memory-specific sequences (right). Rigid units are shown in purple and flexible units are shown in pink. **b** Example of two sequences taken from separate successful memory retrieval trials. Cooler and hotter colors correspond, respectively, to earlier and later in the first sequence, and this unit color mapping was applied to the second sequence in order to emphasize rearrangement of the units from the first sequence to create the second sequence. The blue arrow indicates a unit that was completely rigid in its rank position between the first and second memory, whereas the yellow arrow indicates a flexible unit that rearranged for the second memory (low and high information content respectively). **c** Memory specificity index was significantly greater for flexible units compared to rigid units, indicating that removal of flexible units has a much greater effect on the resultant specificity of spiking sequences corresponding to each memory (two-sided t-test, t(5) = 4.37, *p* = 0.007). (**) indicates *p* < 0.01. Error bars indicate SEM across participants. **d** Average information content for rigid and flexible units in retrieval sequences. Information is displayed as bits/unit, calculated for both spike count (left; two-sided t-test, t(5) = 7.48, *p* < 0.001) and spike latency (right; two-sided t-test, t(5) = 7.97, *p* < 0.001). (***) indicates *p* < 0.001. Error bars indicate SEM across participants. *N* = 6 participants for all statistical tests in this figure.

We were motivated to understand how these rigid and flexible sequence elements, respectively, contribute to episodic memory. We hypothesized that while backbone sequences may represent an average sequence template, flexibility in the sequence space is necessary to form distinct neural representations of different memories (Fig. 4a). Therefore, flexible units should contribute more to the specificity of sequences corresponding to each memory of interest. We found several examples of individual sequences occurring during memory retrieval in which the sequence position of individual units either changed or remained stable across different trials (Fig. 4b). To quantify how much each unit contributed to the memory specificity of each sequence, we defined a memory specificity index for each unit (see “Methods”, Fig. S14). We first measured the true memory specificity by calculating the similarity of the sequences observed during all correct retrieval trials and their corresponding encoding trials and subtracting the average sequence similarity obtained by comparing the retrieval sequences to all non-matching correct encoding trials^12^. This distribution represents an empirical difference between the replay of the specific correct trials and the replay of any correct trial. We subsequently removed each unit from all sequences, recalculated the memory specificity of each trial, and then calculated a t-statistic between the true memory specificity and that computed after removing that unit. This value quantifies how much a given unit contributes to the overall memory specificity of the respective spiking sequences. Across participants, flexible units have a significantly greater memory specificity index than rigid units, indicating that flexible units largely underlie sequence rearrangement during the replay of specific memories (Fig. 4c; t(5) = 4.37, *p* = 0.007).

Given the variability in spike timing observed in the flexible units, we were interested in whether flexible units may encode more information in their spike timing and, conversely, whether rigid units may contain more information in their spike rates. To disentangle this, we directly quantified the information content of single sequences during memory retrieval. We adapted previous methodologies for comparing information conferred by spike counts versus spike latencies^34,35^ (see “Methods”). We focused our measures of information content on the last sequence before vocalization during the retrieval trials, since this sequence usually has the highest replay score^12^. We found that within these sequences, rigid units carry more information in spike counts compared to flexible units (t(5) = 7.48, *p* < 0.001), while flexible units carry more information in spike timing compared to rigid units (t(5) = 7.97, *p* < 0.001; Fig. 4d). These data therefore suggest that the increased memory specificity index observed in flexible units reflects the fact that these units rely on their spike timing to encode information.

## Discussion

Spiking sequences are a fundamental feature of many neural systems and have been suggested to play an important role in information coding in the brain. Although evidence has linked different spiking sequences with different memory representations^12,21,27,36^, underlying neural circuitry has been heavily implicated in the emergence of these empirically measured spiking patterns. Pre-existing neural structure and connectivity has therefore been hypothesized to elicit a constrained space of spiking sequences that may be useful in cognition. Here we demonstrate that backbone sequences can be measured in human cortex and that they persist over time and through different cognitive states. Our data demonstrate that backbone sequences are composed of both rigid and flexible elements, and flexibility around the backbone allows for the formation of memory-specific spiking sequences.

These findings support the hypothesis that pre-existing neural network architecture is a primary determinant of emergent patterns of activity^13,18,20^. In this conceptualization, neural representations of memories are not created de novo, but may be selected from a pre-existing dictionary of items that arise inherently from spontaneous activity. The phenomenon of memory preplay is a natural extension of this framework, with cognition borrowing neural patterns from previously experienced spontaneous activity^16,24^. Our data demonstrating the persistence of backbone sequences through different cognitive states and the preplay of memory relevant spiking sequences during pre-task rest are consistent with these hypotheses.

Our finding that backbone sequences exist in the human temporal lobe cortex is also consistent with recent evidence that both spontaneous and cognitively relevant cortical spiking occupy a constrained subspace of the total possible neural activity. Recent evidence has suggested that communication in the cortex occurs through the transmission of short bouts of cortical activity, or packets, that are highly stereotyped but also contain information about stimulus identity^1^. Sequences of spiking activity may represent these packets, with rigid sequence elements constituting a more general patterning of neural activity that is dependent on the network’s specific structure of external and recurrent connections^28^. Flexible elements within these sequences may add requisite specificity for cognition and therefore determine the span of the backbone sequence space. The variability provided by these flexible elements, and consequently the backbone sequence space, may be important in many behavioral different contexts. For example, the relatively narrow span of sequential activity evoked in the songbird cortical high vocal center is essential for high fidelity replication of a song necessary for mating^37^, but the much larger span of noisier sequences in rodents^21,32^ and humans^12^ may be necessary in order to house a diverse enough repertoire of patterns for higher level memory and cognition. Indeed, noisy replay among a large subspace of similar sequences may be a neural substrate of prediction or creating rich schema from a limited number of episodic memories^38^.

The interplay between rigid and flexible sequence elements also has implications for cortico-hippocampal communication and systems memory consolidation. As our results suggest, memory reactivation involves re-engaging flexible sequence elements with the same timing as in encoding. Particular to the cortex, this may represent a structured read-out of feedforward hippocampal activity^39^, whereby preconfigured ensembles in the cortex are in fact indexed by the hippocampus^40^. On the neurophysiological level, rigid sequence elements are thought to be fast spiking, stronger connected neurons and flexible sequence elements are slow spiking and more weakly connected^18^. Systems memory consolidation in this case may involve a stabilization of these weakly connected flexible sequence elements as memories are consolidated after awake repetition or during sleep. This interpretation would be consistent with the formation of stable cortical modules during memory consolidation as hippocampal memory traces are effectively transferred to cortex in offline states^41^. Future experimental and theoretical studies may investigate the synaptic connectivity of a unit relative to its position in a backbone sequence as well as the advantages of having rigid elements when acquiring and storing new memories.

Taken together, we describe backbone sequences as a basis for ongoing cortical activity in the human brain. These sequences are highly reproducible and persist regardless of cognitive state. In context of our previous work demonstrating memory-specific replay of cortical spiking sequences^12^, the backbone sequence therefore effectively circumscribes a scaffold for flexible memory representations in a paired associates episodic memory task. The backbone sequence may therefore be functionally important in the sense of providing a constrained subset of patterns available for memory in the human cortex. In a larger sense, our data support the growing lines of evidence of structurally predetermined network dynamics that underlie cognition in the human brain. Future studies can further expand upon these findings by directly confirming putative characteristics of networks that generate backbone sequences, which will likely lead to a greater understanding of how the brain organizes and creates neural representations of memory.

## Methods

### Participants

The data presented here were collected in a previous study^12^. 6 participants (2 female; 34.8 ± 4.7 years) with drug-resistant epilepsy underwent a surgical procedure in which platinum recording contacts (PMT Corporation, Chanhassen, MN) were implanted subdurally on the cortical surface as well as deep within the brain parenchyma. For research purposes, we placed one or two 96-channel microelectrode arrays (MEA; 4 × 4 mm, Cereplex I; Blackrock Microsystems, Inc., Salt Lake City, UT) in the anterior temporal lobe (ATL) of each participant in addition to the subdural grid (Fig. S15). MEAs were implanted only in participants with a presurgical evaluation indicating clear seizure localization in the temporal lobe. Hence, although the use of such arrays offer no direct clinical benefit and are purely to advance fundamental knowledge about the human brain, the implant site in the MTG was chosen to fall within the expected resection area. Each MEA was placed in an area of cortex that appeared normal both on the pre-operative MRI and on visual inspection. Across participants, MEAs were implanted 14.6 ± 3.7 mm away from the closest subdural electrode with any ictal or interictal activity identified by the clinical team. Four out of the six participants received a surgical resection which includes the tissue where the MEAs were implanted. One participant had evidence of focal cortical seizure activity and received a localized resection posterior to the MEA site. One participant did not have a sufficient number of seizures during the monitoring period to justify a subsequent resection. Neither participant experienced a change in seizure type or frequency following the procedure, or experienced any noted change in cognitive function. The NINDS Institutional Review Board (IRB) approved the research protocol, and a team of research nurses independent from either the clinical or research teams obtained informed consent from the participants explicitly for the placement of the MEAs and for all research components of this study.

### Paired-associates memory task

Each participant performed a paired associates verbal memory task^12,30,42^. During the study period, participants were sequentially shown a list of word pairs (encoding period) and instructed to remember the novel associations between each pair of words. Later during testing, they were cued with one word from each pair selected at random (retrieval period), and were instructed to say the associated word into a microphone. We designated the rest period as the 2 min before the testing session began. Participants were not specifically instructed to do anything during this period and were waiting for the tester to set up the testing equipment and software.

A single experimental session for each participant consisted of 25 lists, where each list contained six pairs of common nouns shown on the center of a laptop screen. The number of pairs in a list was kept constant for each participant. Words were chosen at random and without replacement from a pool of high-frequency nouns and were presented sequentially and appearing in capital letters at the center of the screen. In order to ensure that memory formation and retrieval were not directly adjacent in the task, study word pairs were separated from their corresponding retrieval cue by a minimum lag of two study or test items. During the study period (encoding), each word pair was preceded by an orientation stimulus (‘+’) that appeared on the screen for 250–300 ms followed by a blank interstimulus interval (ISI) between 500–750 ms. Word pairs were then presented stacked in the center of the screen for 4000 ms followed by a blank ISI of 1000 ms. Following the presentation of the list of word pairs, participants completed an arithmetic distractor task of the form A + B + C = ? for 20 s.

During the test period (retrieval), one word was randomly chosen from each of the presented pairs and presented in random order, and the participant was asked to recall the other word from the pair by vocalizing a response. Each cue word was preceded by an orientation stimulus (a row of question marks) that appeared on the screen for 250–300 ms followed by a blank ISI of 500–750 ms. Cue words were then presented on the screen for 4000 ms followed by a blank ISI of 1000 ms. Participants could vocalize their response any time during the recall period after cue presentation. We manually designated each recorded response as correct, intrusion, or pass. A response was designated as pass when no vocalization was made or when the participant vocalized the word ‘pass’. During pass trials where no vocalization was present, we assigned a response time by randomly drawing from the distribution of correct response times during that experimental session. We defined all intrusion and pass trials as incorrect trials. A single experimental session contained 150 total word pairs. Each participant completed between 1–3 sessions (2.2 ± 0.3 per participant).

### Identification of single units

We identified single units as reported previously^12,30,31^. Briefly, microelectrodes were arranged in a 10 × 10 grid with each electrode spaced 400 μm apart and extending 1.5 mm into the cortical surface (1.0 mm for one participant). Post-operative paraffin blocks of the resected tissue demonstrated that the electrodes extended approximately halfway into the 3-mm-thick gray matter. We digitally recorded microelectrode signals at 30 kHz using a Cerebus acquisition system (Blackrock Microsystems NeuroPort Central Suite, version 7.0.3.0), with 16-bit precision and a range of ±8 mV. To extract neuronal spiking activity, we re-referenced each electrode’s signal offline by subtracting the mean signal of all the electrodes in the MEA, and then used a second-order Butterworth filter to bandpass the signal between 0.3 to 3 kHz. We extracted micro-scale local field potential (LFP) signals in the same manner, but instead used a 500 Hz low pass filter. Using a spike-sorting software package (Plexon Offline Sorter, Dallas, TX, USA), we identified spike waveforms by manually setting a negative or positive voltage threshold depending on the direction of putative action potentials. The voltage threshold was set to include noise signals used in calculating unit isolation quality (see below). Waveforms (duration, 1.067 ms; 32 samples per waveform) that crossed the voltage threshold were stored for spike sorting. Spike clusters were manually identified by viewing the first two principal components, and the difference in peak-to-trough voltage (voltage versus time) of the waveforms. We manually drew a boundary around clusters of waveforms that were differentiable from noise throughout the experimental session. In this manner, we identified a total of 989 putative single units across all sessions (average of 72.2 ± 20.7 units per participant). The average spike rate across all units was 2.5 ± 0.6 Hz.

Due to variability in the signal quality across recordings and the subjective nature of spike sorting, we quantified the quality of each unit by calculating an isolation score and signal to noise ratio (SNR). The isolation score quantifies the distance between the spike and noise clusters in a 32-dimensional space, where each dimension corresponds to a sample in the spike waveform. The spike cluster consisted of all waveforms that were classified as belonging to that unit, and the noise cluster consisted of all waveforms that crossed the threshold that were not classified as belonging to any unit. The isolation score is normalized to be between 0 and 1, and serves as a measure to compare the isolation quality of all units across all experimental sessions and participants. Across participants, the mean isolation score for all units was 0.93 ± 0.1.

In addition to isolation quality, we computed the SNR for each unit using the following equation:

1 $${{{{{{\rm{SNR}}}}}}}=\frac{{V}_{{peak}}-{V}_{{trough}}}{{Noise}*C}$$

where *V*_*peak*_ and *V*_*trough*_ are the maximum and minimum voltage values of the mean waveform, and *C* is a scaling factor (set as 5). To obtain *Noise*, we subtracted the mean waveform from each individual waveform for each identified unit, concatenated these waveform residuals, and then computed the standard deviation of this long vector. Therefore, the noise term quantifies the within-unit variability in waveform shape. Across participants, the mean SNR for all units was 1.71 ± 0.12.

### Extraction of backbone sequences

To calculate the backbone sequence from a given epoch of time, we adapted previously reported methods for determining the average spike latencies between each unit and the population activity^21,32^. The backbone sequence allows for the calculation of an average sequential order of firing for neurons, even though each individual burst within that period of time typically has a different specific order. We first cross-correlated the spike train of each unit with the summed activity from all other simultaneously recorded units. We chose a ±75 ms cross-correlation window based on previous literature for cortical spontaneous and evoked sequences^1^ as well as empirical evidence from spiking sequences in human cortex^12^. The resulting cross-correlogram was smoothed with a Gaussian kernel (*σ* = 10 ms) in order to determine the temporal index of the peak. Units were then arranged in time based on their respective maximum cross-correlogram peaks in order to form an average backbone sequence for the time epoch of interest. We note then that individual spikes can appear out of order with respect to the overall backbone sequence since we only enforce that the cross-correlation maxima are strictly ordered in time. For visualization purposes, we normalized the cross-correlogram for each unit between 0 and 1 by dividing by the largest value so that units were comparable regardless of baseline activity levels. For most analyses in this study, we evaluate backbone sequences extracted from 2 min non-overlapping epochs over 40 min of an episodic memory task (i.e., Fig. 1e). In one participant, one of the recording sessions was slightly shorter than 40 min, and therefore the last 2 min epoch was not used for subsequent analyses in that participant.

### Identification of burst events and sequences

We identified burst events as reported previously^12^. We calculated the instantaneous population spike rate by convolving a Gaussian kernel (*σ* = 25 ms) across the spike rasters for each unit. We obtained an average spike rate over each trial for each unit, and then averaged across all units to obtain an average population spike rate for each trial. We used the distribution of trial averaged spike rates to determine the mean and standard deviation of the population spike rate for each experimental session. Within each session, we then defined the burst event threshold as three standard deviations above the mean of the distribution of trial averaged spike rates. We identified burst events as an event exceeding this threshold for at least 25 ms (Fig. S16). We assigned the time point with the highest population spike rate within each burst event as the temporal index of that burst event, and we defined the burst event window as the time period ±75 ms from this index. In order to avoid extracting sequences from overlapping burst events that likely indicated the same event, we removed any event that was within 150 ms of another by removing the event with the lower spiking rate. Events that extended outside of the trial window boundaries (e.g., after start of vocalization) were excluded in order to retain only trial relevant spiking activity.

Once burst events had been identified, we extracted sequences from the units that fired during each event. We identified these sequences by determining the temporal order of maximal firing rate of each unit within the burst event time window. We calculated the rates of each unit with the same Gaussian kernel above and assigned each unit a position within the extracted sequence based on when its maximum firing rate occurred within the burst event window. We based calculations of sequence similarity between pairs of burst events on the sequences of maximal firing in each burst event identified in this manner.

### Quantification of sequence similarity

We quantified sequence similarity using the matching index^9,12^ (Fig. S17). For a given sequence of length *N*, a total of *N*(*N*−1)/2 pairs can be assessed between neurons occurring in different positions within the sequence. For a second sequence that is to be matched to the first, we define *m* as the number of pairs of neurons that fire in the same order as in the first sequence, and *n* as the number of pairs of neurons that fire in the opposite order. The matching index (MI) is then defined as:

2 $${{{{{{\rm{MI}}}}}}}=(m-n)/(m+n)$$

MI is bounded between −1 and 1, indicating exactly reverse and forward replay, respectively, of the second sequence of interest relative to the original sequence. We determined the significance for how similar a given sequence pair is by randomly rearranging the temporal positions of units in each sequence 1000 times, calculating the MI for each permutation, and comparing the true MI to the shuffled distribution^9^.

### Classification of rigid and flexible single units

To assess the rigidity of a unit’s position in a given backbone sequence, we first calculated the normalized sequence rank of the unit of interest in each time bin of a given session (e.g., Fig. 3b). For example, a unit that appeared second in a sequence of 10 units would be given a normalized rank of 0.2. In this manner, each unit was assigned a distribution of normalized sequence ranks, and we subsequently calculated the variance of this distribution for a true measurement of the consistency of the sequence rank over time. To create a null distribution for comparison against this true measurement, we randomly shuffled the positions of every unit 500 times and calculated the variance of normalized sequence ranks for each shuffle. We z-scored the true variance value against the shuffled distribution, allowing for a normalized metric of rigidity against a null distribution that is comparable across participants. Under this paradigm, more rigid units have a more negative z-score because the true variance of sequence rank should be smaller than the null distribution. We therefore classified a unit as rigid or flexible if the given z-score was <−1 or >1, respectively. This threshold is relatively arbitrary, however, so we reproduced all of the main results using different thresholds (Fig. S12).

For an additional confirmation of the validity of our methods, we reproduced the main results of the study by using an alternative metric of rigidity within a sequence (Fig. S13). We first hypothesized that a rigid unit would likely contribute substantially more to backbone sequence similarity over time than a flexible unit. We therefore shuffled the sequence position of each unit 500 times and recalculated the average similarity between the resting backbone sequence and backbone sequences extracted later in the session (i.e., Fig. 1e). We then z-scored the true average sequence similarity value against the average value of each permutation to create a normalized value that is comparable across participants. In this way, more rigid units are expected to have a more positive z-score because the true sequence similarity should be higher compared to sequences where the position of a rigid unit has been shuffled randomly. We classified a unit as rigid or flexible if the given z-score was >1 or <−1, respectively, and were able to reproduce all of the main results (Fig. S13).

### Quantification of contribution to memory specificity of single units

We were motivated to understand if rigid and flexible units contributed differentially to the memory specificity of individual spiking sequences. We first calculated the true memory specificity of each trial by calculating the true sequence similarity between correct retrieval and encoding of matching trial pairs and subtracting the average values obtained after shuffling the trial labels of all correct trials^12^. In this way, the true memory specificity represents the difference in measured sequence similarity between a specific retrieved memory (i.e., matching encoding and retrieval trials) and any correctly retrieved memory. This generates a distribution of true memory specificity values across all correct trials. To investigate how a single unit in question contributed to memory specificity, we removed each unit and recalculated memory specificity for each trial as outlined above. We quantify the memory specificity index of a given unit as the t-statistic comparing the true distribution of memory specificity values for all correct trials compared to the same distribution calculated after removing the unit. Under this paradigm, if a unit contributes greatly to memory specificity, its removal would on average decrease memory specificity across trials, and the t-statistic would be positive because the true distribution would be greater than the distribution generated with the unit removed (Fig. S14).

### Quantification of information content in sequences

To explicitly quantify the information content contained in spiking sequences, we adapted previously reported techniques^34,35^. We chose to analyze the last individual sequence before vocalization given previous evidence that this sequence contained the highest replay values during memory retrieval^12^. The information content of an individual neuron’s spiking activity in response to different stimuli is given by the following equation:

3 $$I(S {{{{{\rm{;}}}}}} \, R)={\sum }_{r,s}P(s)P(r{{{{{\rm{|}}}}}}s){lo}{g}_{2}\frac{P(r{{{{{\rm{|}}}}}}s)}{P(r)}$$

where *P*(*s*) is the probability of a stimulus, *P*(*r*|*s*) is the probability of a response ‘*r*’ given a particular stimulus ‘*s*’, and *P*(*r*) is the probability of a response ‘*r*’. In practice, *P*(*s*) is defined as one divided by the total number of stimuli presented per session, which reflects a uniform distribution of stimuli since we only present each unique stimulus once. To determine the information contained in the number of spikes a given unit exhibits, we define *P*(*r*) as simply the fraction of all sequences that contain a particular spike count for that unit. Likewise, to determine how much information is contained in the spike latencies, we define *P*(*r*) as the fraction of sequences that contain that particular spike latency for that unit. To allow for some temporal jitter in this metric, we binned the 150 ms sequence duration into 10 equally spaced bins and determined the likelihood of a neuron firing in each of these bins. In this case, therefore, a latency of 5 ms for example may be assigned to the same bin as a latency of 7 ms. In determining the information content based both on spike count or spike latency, *P*(*r*|*s*) in practice is equal to one because our sample space is one unique sequence per one unique stimulus. After calculating information content per unit in each sequence, we averaged separately over all rigid and flexible units to obtain values of information content in bits/unit.

### Intracranial EEG (iEEG) recordings

We collected intracranial EEG (iEEG) data from a total of 716 subdural and depth recording contacts (119 ± 16.0 per participant). Subdural contacts were arranged in both grid and strip configurations with a contact radius of 1.5 mm and inter-contact spacing of 10 mm. These contacts could lie along medial temporal lobe (MTL) structures including the parahippocampal gyrus and entorhinal cortex. We designated an electrode as residing in the MTL if its placement was medial to the collateral sulcus, excluding the uncus. Contact localization was accomplished by co-registering the post-op CTs with the post-op MRIs using both FSL Brain Extraction Tool (BET) and FLIRT software packages and mapped to both MNI and Talairach space using an indirect stereotactic technique and OsiriX Imaging Software DICOM viewer package. The resulting contact locations were subsequently projected to the cortical surface of a Montreal Neurological Institute N27 standard brain (Fig. S15)^43^. Pre-operative MRIs were used when post-operative MR images were not available. We identified the location of each MEA on a surface reconstruction created using each participant’s pre-operative T1 weighted MRI (FreeSurfer, http://surfer.nmr.mgh.harvard.edu). Individual participant reconstructions were co-registered with a standard template brain, and the locations of each participant’s MEA were visualized on the template brain.

Depending on the amplifier and the discretion of the clinical team, iEEG signals were sampled at 1000 or 2000 Hz. Data were acquired using Nihon Khoden’s EEG data acquisition software (NeuroWorkbench version 7-02). For clinical visual inspection of the recording, signals were referenced to a common contact placed subcutaneously, on the scalp, or on the mastoid process. The recorded raw iEEG signals used for analyses were referenced to the system hardware reference, which was set by the recording amplifier (Nihon Kohden, Irvine, CA) as the average of two intracranial electrode channels. We re-referenced these raw signals using bipolar referencing in order to mitigate any effects of volume conduction or any biases introduced by the system hardware reference^42^. All recorded traces were resampled at 1000 Hz, and a fourth-order 2 Hz stopband Butterworth notch filter was applied at 60 Hz to eliminate electrical line noise.

### Ripple detection

We detected ripple events as reported previously^42^. We first bandpass filtered the iEEG signal in the ripple band (80–120 Hz) using a second-order Butterworth filter, and then applied a Hilbert transform to extract the instantaneous amplitude within that band. We selected events where the Hilbert envelope exceeded 2 standard deviations above the mean amplitude of the filtered traces. Only events that were at least 25 ms in duration and had a maximum amplitude greater than 3 standard deviations were retained as ripples for analysis. We joined adjacent ripples that were separated by <15 ms. We identified every ripple that satisfied these criteria in every electrode contact, and assigned each such identified ripple a start time index and an end time index. The difference between them defined the duration of each ripple. The detected macro-iEEG ripples in our data set had an average peak frequency of 86.7 ± 1.6 Hz (Fig. S18). The average rate of ripple occurrence per electrode was 0.20 ± 0.02 Hz, consistent with previous reports^42^.

Importantly, high-frequency activity can be associated with epileptiform activity in addition to cognitive processes. Therefore we implemented several measures to provide the most conservative sampling of non-pathological signals possible. We implemented a previously reported automated trial and electrode rejection procedure based on excessive kurtosis or variance of iEEG signals^30,42^. We calculated and sorted the mean iEEG voltage across all trials, and divided the distribution into quartiles. We identified trial outliers by setting a threshold, Q3 + w∗(Q3 − Q1), where Q1 and Q3 are the mean voltage boundaries of the first and third quartiles, respectively. We empirically determined the weight w to be 2.3. We excluded all trials with mean voltage that exceeded this threshold. The average percent removed across all sessions in each participant due to either system-level noise or transient epileptiform activity was 4.6 ± 3.4% of all trials.

In addition, system level line noise, eye-blink artifacts, sharp transients, and interictal epileptiform discharges (IEDs) can be mistakenly characterized as ripples after high pass filtering. We therefore implemented a previously reported automated event-level artifact rejection^42^. We calculated a z-score for every iEEG time point based on the gradient (first derivative) and amplitude after applying a 250 Hz high pass filter (for identification of epileptogenic spikes). Any time point that exceeded a z-score of 5 with either gradient or high-frequency amplitude was marked as artifactual, and 125 ms before and after each identified time point was also classified as an artifact. We visually confirmed that the above methodology reliably identified IEDs and high-frequency oscillations associated with IEDs (ripple on spike waveforms)^42^. We excluded all IEDs and all such pathologic ripples from our analyses. The remaining ripples that we retain for our analyses therefore occur without an associated IED, and we note that this explicitly enforces that ripples and IEDs are necessarily non-overlapping in our final data set.

We performed a non-parametric clustering-based procedure to determine the temporal indices of significant preplay following MTL ripples^12^ (Fig. 2d). The clustering procedure identifies contiguous temporal epochs exhibiting significant differences between trial types with the null hypothesis that across participants, each epoch showed no difference for preplay of correct versus incorrect trials. For each time epoch, we computed the true t-statistic and *p*-value across participants between correct and incorrect trials. We then randomly permuted the participant-specific averages (correct versus incorrect), which in practice translates to randomly reversing the sign of the difference within each participant and recomputing the mean difference across participants. For n participants, this results in an empiric distribution of 2^n^ possible mean differences that are all equally probable under the null hypothesis. We generated the empiric distribution from 500 permutations for every epoch and calculated t-statistics for each of the permuted epochs. The *p*-value for each individual epoch in the true case, however, does not take into account the multiple comparisons that are made in time (across epochs).

To correct for multiple comparisons across epochs, we identified clusters containing epochs that were adjacent in time that exhibited a significant difference between trial types (where in each epoch, *p* < 0.05). For each cluster of significant epochs identified in the true and permuted cases, we defined a cluster statistic as the sum of the t-statistics within that temporal cluster. We retained the maximum cluster statistic during each of the 500 permutations to create a distribution of maximum cluster statistics. We assigned *p*-values to each identified cluster of the true data by comparing its cluster statistic to the distribution of maximum cluster statistics from the permuted cases. Clusters were determined to be significant if their *p*-value calculated in this manner was <0.05.

### Reporting summary

Further information on research design is available in the Nature Portfolio Reporting Summary linked to this article.

### Supplementary information

Supplementary Information

Reporting Summary

### Source data

Source Data

## Data Availability

The data that support the findings of this study are available for public download at https://research.ninds.nih.gov/zaghloul-lab/downloads. Source data are provided with this paper.

## Code availability

Except where otherwise noted, computational analyses were performed using custom written MATLAB (MathWorks) scripts. The custom MATLAB scripts used for analysis are available upon request.
