# Supplementary material for: Backbone spiking sequence as a basis for preplay, replay, and default states in human cortex
Source: Nat Commun. 2023 Aug 7;14:4723. doi: 10.1038/s41467-023-40440-5 (PMC10406814; doi:10.1038/s41467-023-40440-5)
Supplement: Supplementary file 1 — Supplementary Information [file 41467_2023_40440_MOESM1_ESM.pdf]

Supplementary Figures

Fig. S1

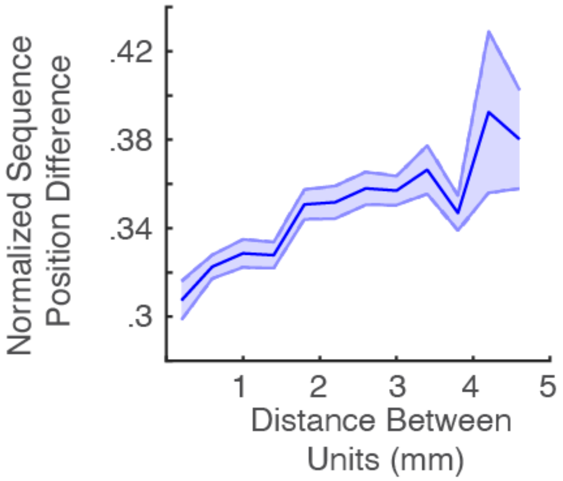

Spatial relationship between units and their relative sequential position. We plotted the physical distance between each pair of units on the MEA against the normalized backbone sequence position difference for those same units. This relationship is significantly positively correlated across all participants ($r = 0.07 \pm 0.02$; $N = 6$ participants, two-sided t-test, $t(5) = 3.03$, $p = .03$). Error bars indicate SEM across participants.

Fig. S2

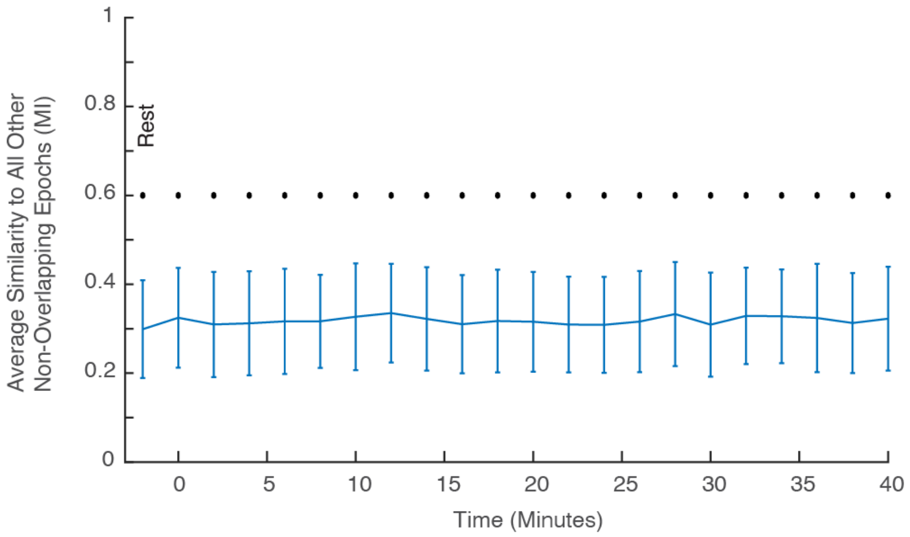

Replication of backbone sequence consistency results with a different sequence comparison methodology. The backbone sequence across patients is consistent across the behavioral task regardless of the cognitive state at any point in time. Sequence similarity over time is calculated here as the average similarity between a given 2 minute non-overlapping bin and all other bins during the entire behavioral session. Error bars represent SEM across participants. Significance of each bin is denoted as a black dot (N = 6 participants, one sided t-test against a null of 0), and testing for multiple comparisons is corrected by FDR (q = 0.05).

Fig. S3

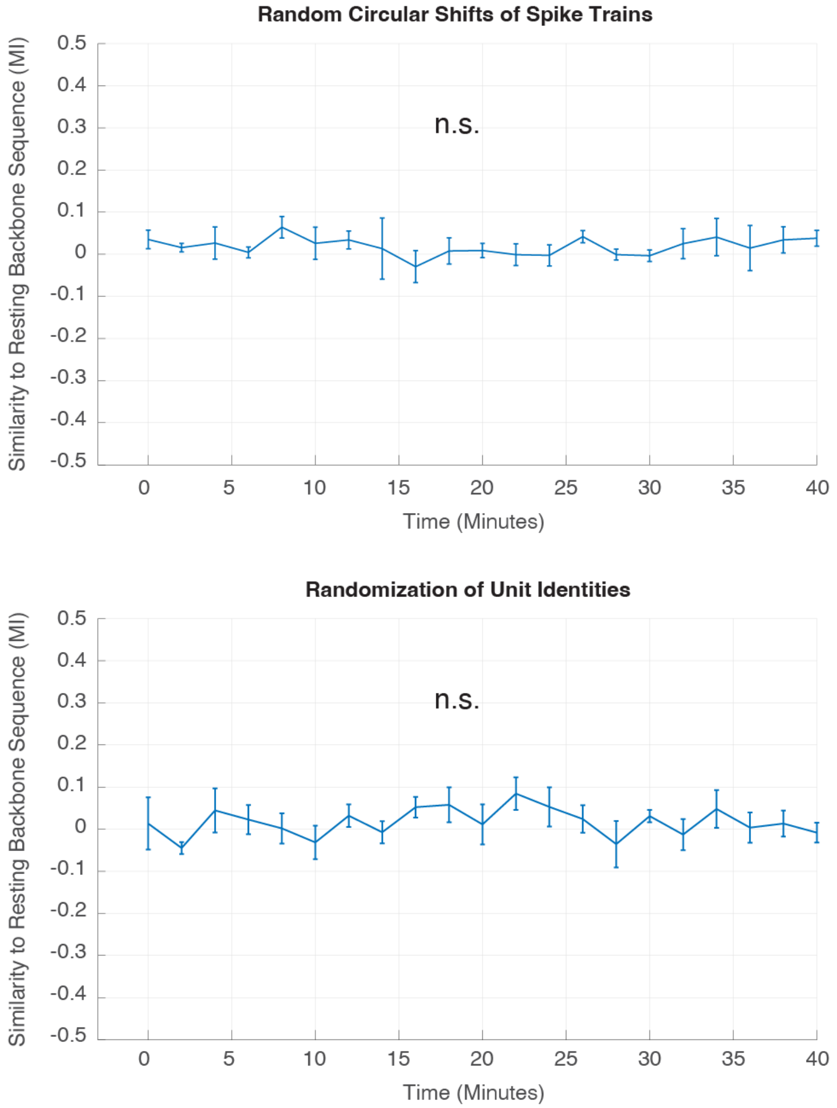

Surrogate distributions for backbone sequence stability. To provide control analyses for the backbone sequence stability plots (Fig. 1e), we shuffled the data in two separate ways. First, we circularly shifted the spike trains of each unit before re-calculating the backbone sequence for each epoch (top). Secondly, we randomized the identities of each unit within each backbone sequence (bottom). Neither of these distributions has significantly similar epochs to the resting backbone sequence (N = 6 participants, one sided t-test against a null of 0, corrected for multiple comparisons by FDR (q = 0.05). Error bars indicate SEM across participants.

Fig. S4

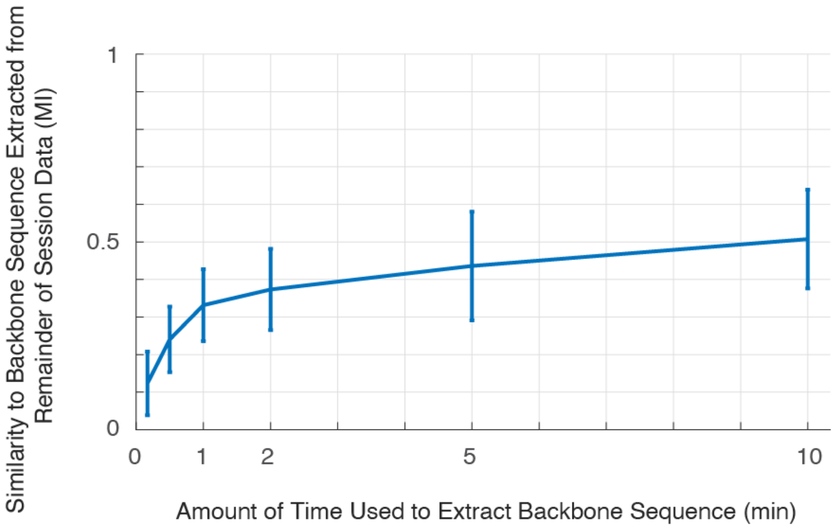

Stability of backbone sequence as a function of duration of time. We were interested in testing how the amount of time used for backbone extraction affected the final observed sequence. We extracted backbone sequences using only 10 seconds, 30 seconds, 1 minute, 2 minutes, 5 minutes, and 10 minutes and subsequently calculated the similarity of these sequences to those extracted from the remainder of the session data. We confirmed the prediction that using less time gives a less accurate estimate of the underlying backbone sequence and that a 2 minute bin size is a reasonable length of time for binning the behavioral task for subsequent analyses. Error bars indicate SEM across participants.

Fig. S5

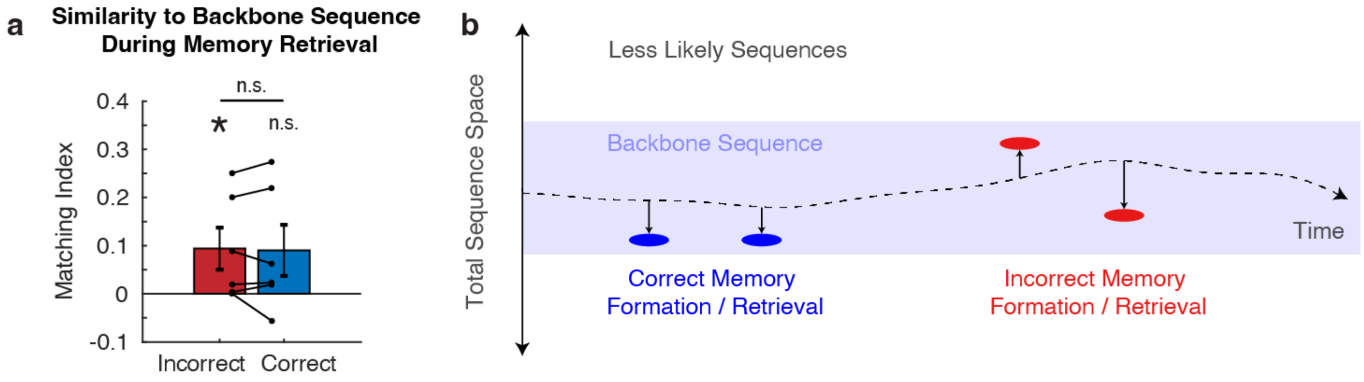

Similarity to backbone sequence is not significantly modulated during memory retrieval. a) We calculated the average similarity between the resting backbone sequence and retrieval sequences in the 1000ms prior to vocalization. There is no significant difference between correct and incorrect retrieval trials (N = 6 participants, two-sided t-test, $t(5) = -0.27$, $p = 0.80$). Error bars indicate SEM across participants. b) We conceptualize this finding as the backbone sequence being overall similar to both correct and incorrect sequences (as in Fig. 2b). Correct memory then involves selective traversal of the total sequence space back to the original point of encoding, which does not necessarily involve a more dissimilar sequence from the backbone.

Fig. S6

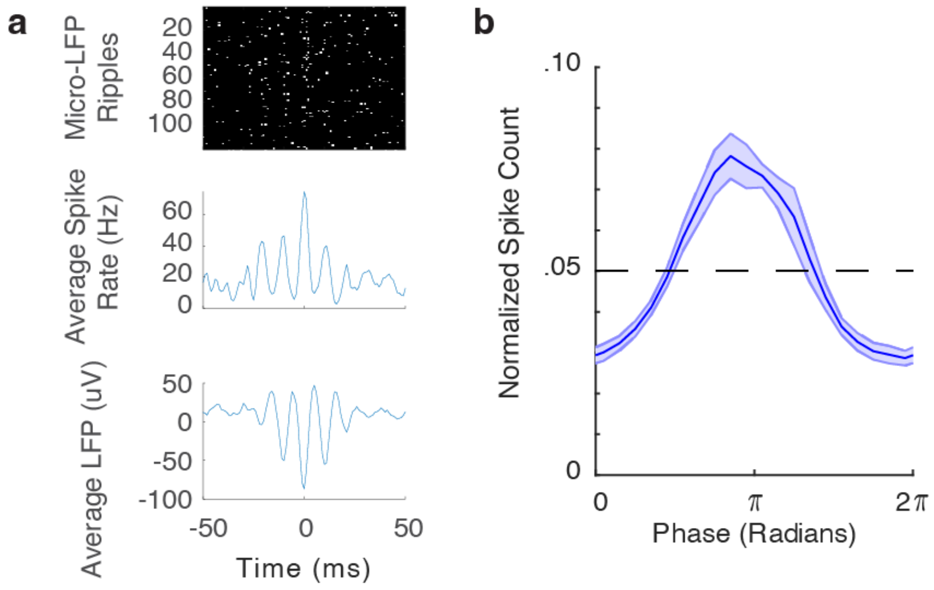

Single unit spikes are preferentially coupled to the ripple phase trough. a) A representative single channel spike raster locked to the maximum trough of each ripple oscillation recorded in that channel (top), with average spike rate (middle) and LFP (bottom) across all ripple events. t = 0 indicates the trough of the detected ripple oscillation. b) Average phase preference of spike with respect to ripple phase. The dotted line indicates the uniform distribution that would be expected by chance given the number of histogram bins. Error bars represent SEM across all participants.

Fig. S7

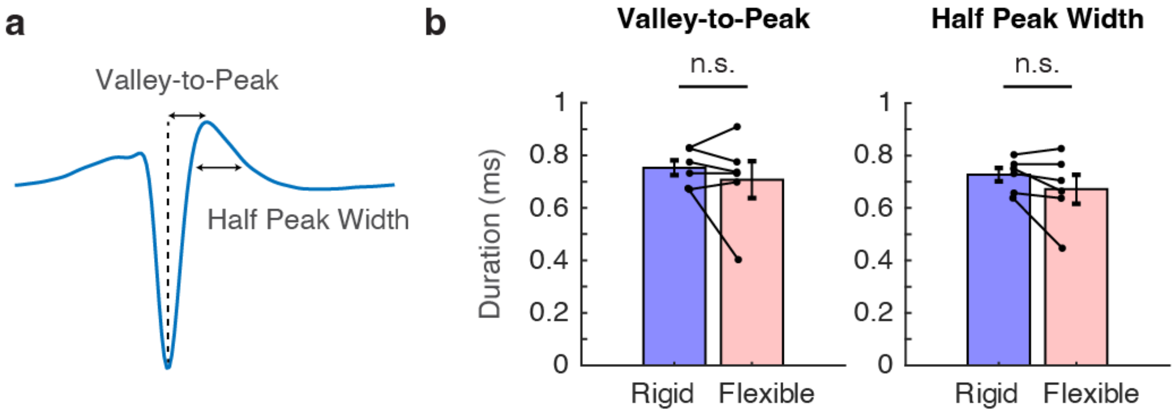

Waveform characteristics of rigid and flexible units are not significantly different. a) We measured the valley-to-peak and half peak width of each unit in our data set in an effort to distinguish excitatory and inhibitory units [33]. b) The across participant averages for each metric are not significantly different between rigid and flexible units (valley-to-peak: two-sided t-test, $t(5) = 0.84$, $p = 0.44$; half peak width $t(5) = 1.58$, $p = 0.18$). Error bars indicate SEM across participants. N = 6 participants for all statistical tests in this figure.

Fig. S8

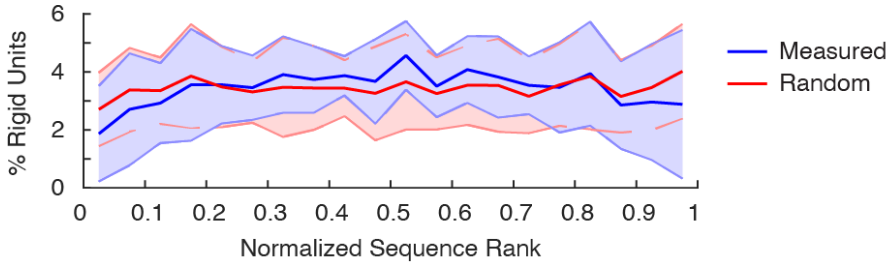

Sequence position of rigid units is not significantly different than chance. We calculated the fraction of observed rigid units in 20 evenly spaced bins of normalized sequence rank (blue) and compared this against the same distribution calculated after randomizing unit identities (red). Shaded error bars in either case represent the SEM across all participants. No bins of the empiric distribution are significantly different than chance after correction for multiple comparisons, FDR (q = 0.05).

Fig. S9

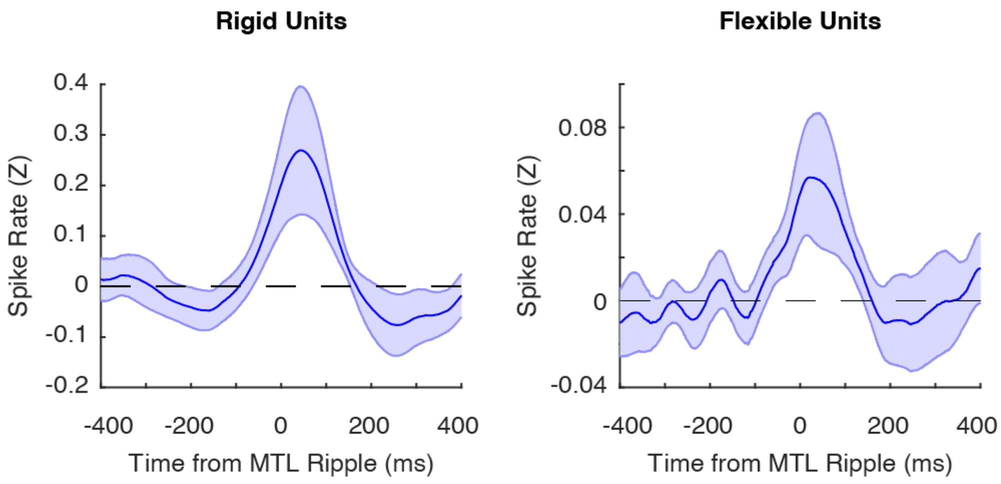

Medial temporal lobe ripples precede increased firing rates of both rigid and flexible units. We calculated the instantaneous spike rate of rigid (left) and flexible (right) cortical units in relationship to MTL ripples. t = 0 indicates the onset of the MTL ripple. Error bars indicate the SEM across all participants.

Fig. S10

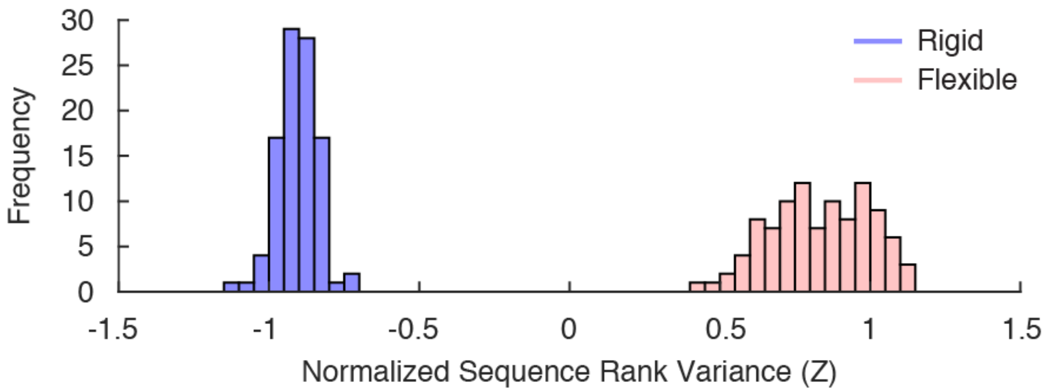

Underlying spike rates do not bias measurements of rigidity. Given our findings of rigid units having much higher firing rates than flexible units, we were concerned that the underlying spike rate of a given unit could artifactually bias a unit towards being more rigid within a backbone sequence. We consequently performed a procedure where the spike rates of rigid units were downsampled in order to match the spike rates of flexible units, and we recalculated the normalized sequence rank variance for each unit based on its original classification as rigid or flexible. This procedure was accomplished by randomly removing spikes from the raster of each unit such that the total number of spikes was equal between the respective rigid and flexible units in question. Because this process is random, we repeated this procedure for 100 permutations in order to generate the above distributions. We plot the average Z-scored normalized sequence rank variance across participants for each permutation. We conclude that rigidity of a given unit within a backbone sequence is not due to its average spike rate. The final average spike rate across all permutations and participants was 0.6 Hz for both rigid and flexible units.

Fig. S11

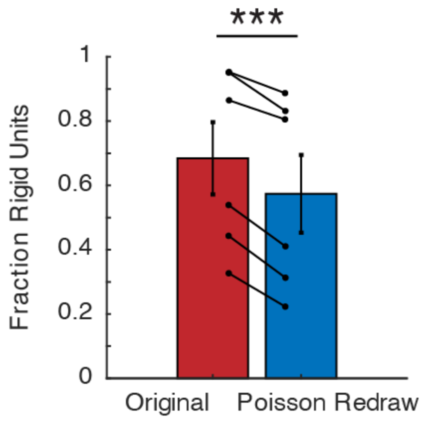

Control analysis showing that underlying spike rates do not bias measurements of rigidity. We jittered the spike trains of each unit by treating each spike train as an inhomogeneous Poisson process. We first calculated the instantaneous spike rate of each unit by convolving with a unit-density Gaussian kernel (σ = 10ms) and then used this rate to generate Bernoulli random variables to determine the generation of spikes in each time bin. In this way, the spike times for each unit are randomized in a Poisson fashion, but the overall average spike counts are the same as the original. We found that the fraction of rigid units calculated in this way uniformly decreases across all patients (N = 6 participants, two-sided t-test, $t(5) = -8.02$, $p < .001$), indicating that the timing of spikes from rigid units determines rigidity as opposed to the mean spike rate. Error bars indicate SEM across participants.

Fig. S12

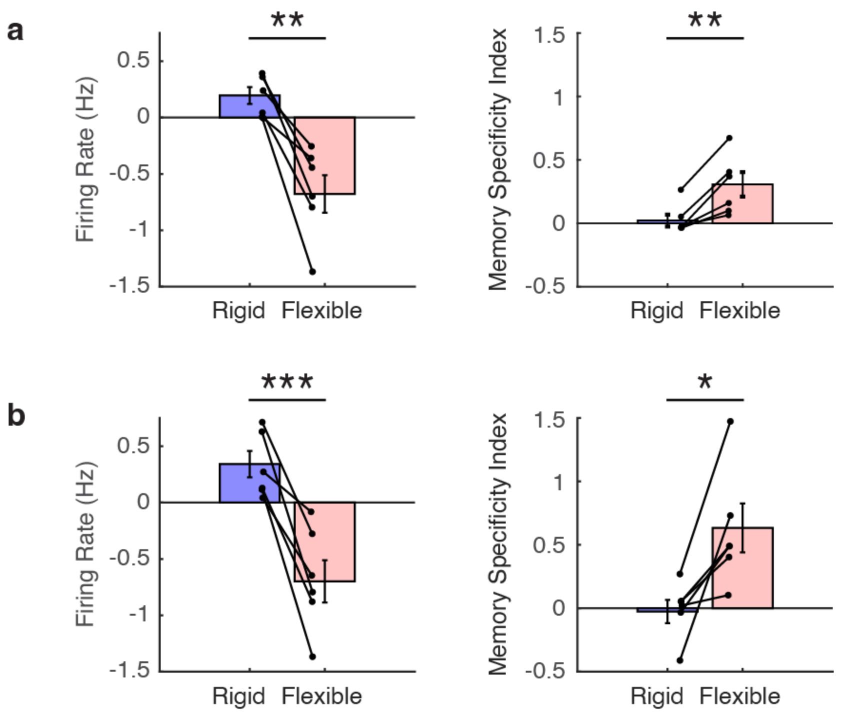

Replication of main results with different thresholds for classification of rigid and flexible units. Because the choice of threshold for the rigid and flexible units is relatively arbitrary, we reproduced the main results by choosing separate thresholds for the Z-scored normalized sequence rank variance of each unit. a) First, for a more liberal threshold of $Z < -0.5$ for rigid units and $Z > 0.5$ for flexible units, we found that firing rate was still higher for rigid units ($t(5) = 5.74$, $p = 0.002$), and the memory specificity index was higher for flexible units ($t(5) = 4.52$, $p = 0.006$). b) We also reproduced these findings for a more conservative threshold of $Z < -2$ for rigid units and $Z > 2$ for flexible units (firing rates: $t(5) = 8.97$, $p < 0.001$; memory specificity index: $t(5) = 3.59$, $p = 0.016$). Two sided t-tests were used for all plots. Error bars for all plots indicate SEM across participants. N = 6 participants for all statistical tests in this figure.

Fig. S13

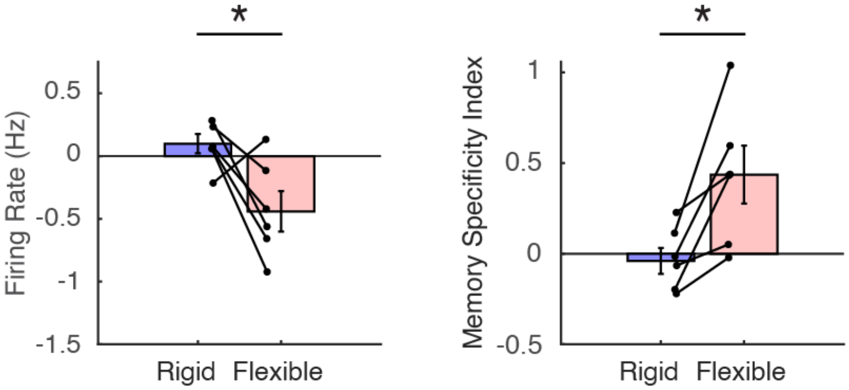

Replication of main results with an alternative measure of rigidity. We alternatively measured rigidity of each unit by randomly shuffling its position within each backbone sequence and recalculating the average sequence similarity to the resting backbone sequence over time (ie. Fig. 1e). We hypothesized that shuffling more rigid units should decrease the average sequence similarity, whereas shuffling shuffling flexible units would have much less of an effect. The z-score of the true sequence similarity value against 500 shuffles then allows for a normalized value of rigidity for classification of units as rigid or flexible. We reproduced the main results of our study in this fashion, with rigid units demonstrating a significantly higher spike rate than flexible units ($t(5) = 2.59$, $p = 0.0487$) and flexible units having a higher memory specificity index ($t(5) = 3.52$, $p = 0.0170$). N = 6 participants and two sided t-tests were used for all statistical tests. Error bars indicate SEM across participants.

Fig. S14

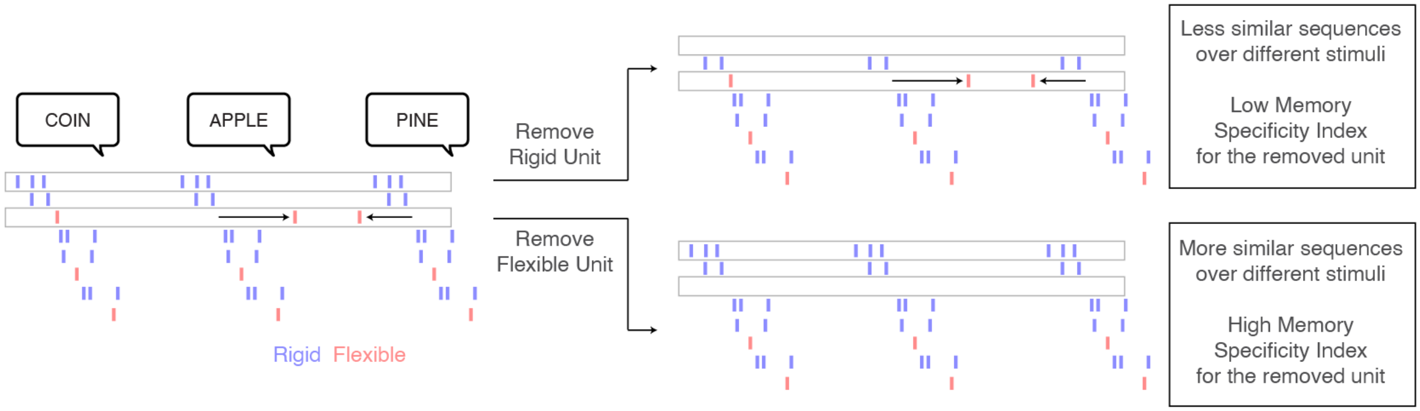

Conceptual diagram for memory specificity index. The memory specificity index quantifies the change in similarity of sequences associated with different stimuli after removing a given unit. In the true data, each sequence is hypothesized to be specific to the item encoded and retrieved from memory. The similarity between sequences from a retrieval trial and its respective encoding trial should therefore be greater than the similarity between that retrieval sequence and all other non-matching encoding trials. We computed this difference for every correct trial, generating a distribution of true values for memory specificity across correct trials. To calculate how much each unit contributes to memory specificity, we systematically removed each unit and repeated this analysis. A rigid unit, for example, should occupy a similar sequence position for many different stimuli. Hence, removing a rigid unit will have the effect of reducing the similarity between any one correct trial and all other correct trials. The memory specificity computed in this case will be higher than in the true case since the distribution of shuffled comparisons will have overall lower similarity. Therefore the difference in memory specificity between the true data and the data with the unit removed, which we define as the memory specificity index, will decrease (top row). Conversely, a flexible unit should occupy different sequence positions in different trials. Removing a flexible unit will therefore have the effect of causing the shuffled pairings to appear more similar to one another, which in turn will cause the memory specificity in this case when removing the flexible unit to be lower than in the true case. Taking the difference between memory specificity between the true data and the data after removing this unit in this example will be positive, hence generating a higher memory specificity index for this unit (bottom row).

Fig. S15

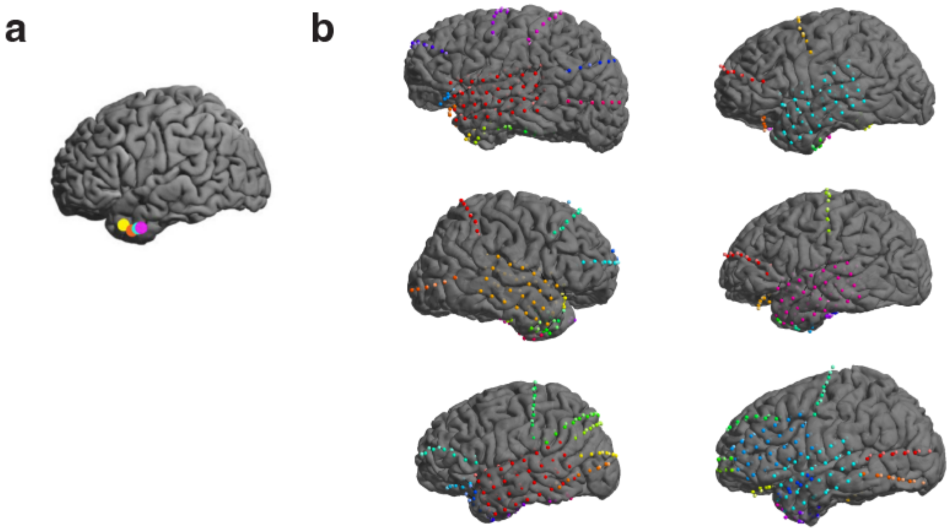

Electrode positions for all participants. a) MEAs were implanted in the anterior temporal lobe of all 6 participants in the expected site of resection (one R sided MEA is not shown). b) Subdural macro-iEEG contact locations for each participant. Different colors indicate separate electrode grids or strips. Figures were generated as previously described [43].

Fig. S16

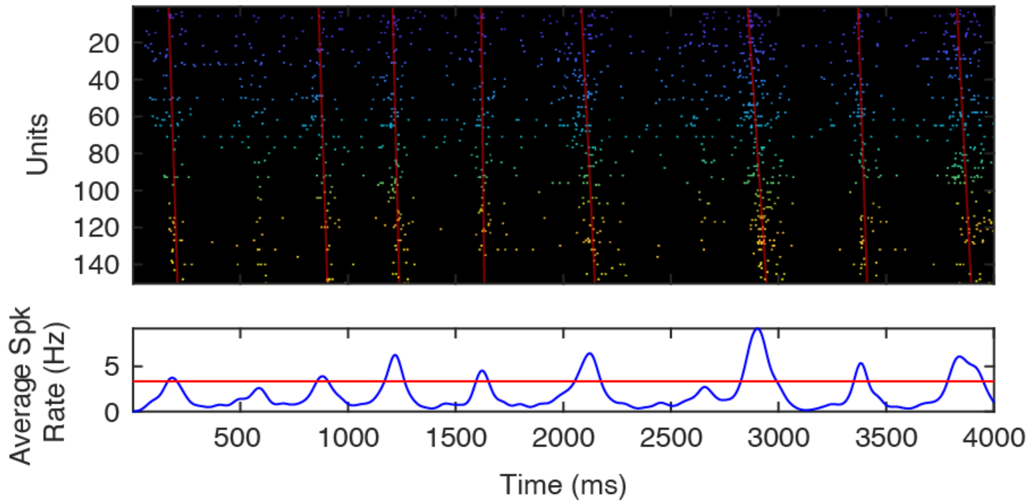

Burst detection and individual sequence extraction. An example spike raster is shown for a representative encoding trial in a single participant (top). Colors represent the average temporal ordering during encoding with cooler and warmer colors representing earlier and later in the sequence respectively. Red lines indicate the linear regression through the maximum spike rate times of all units within each detected burst event. Instantaneous spike rates are shown below the raster (blue) with the burst event threshold for that session (red).

Fig. S17

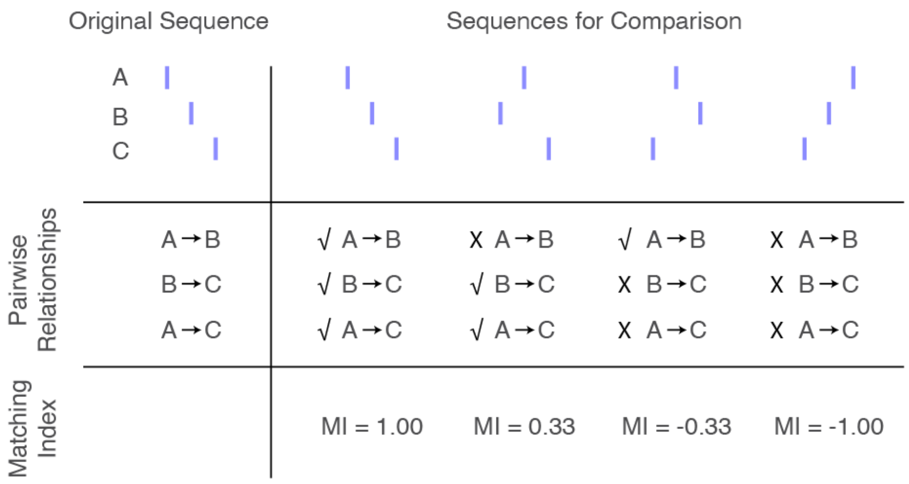

Conceptual diagram for matching index. The matching index is calculated according to previously reported methodologies [9], and is equal to (m - n) / (m + n) where m is the number of pairwise relationships that are preserved in the sequence for comparison, and n is the number of pairwise relationships that occur in the opposite order. Therefore MI is equal to 1 when the sequence is exactly the same (eg. exact forward replay), and the MI is equal to -1 when the sequence is exactly the opposite (eg. exact reverse replay).

Fig. S18

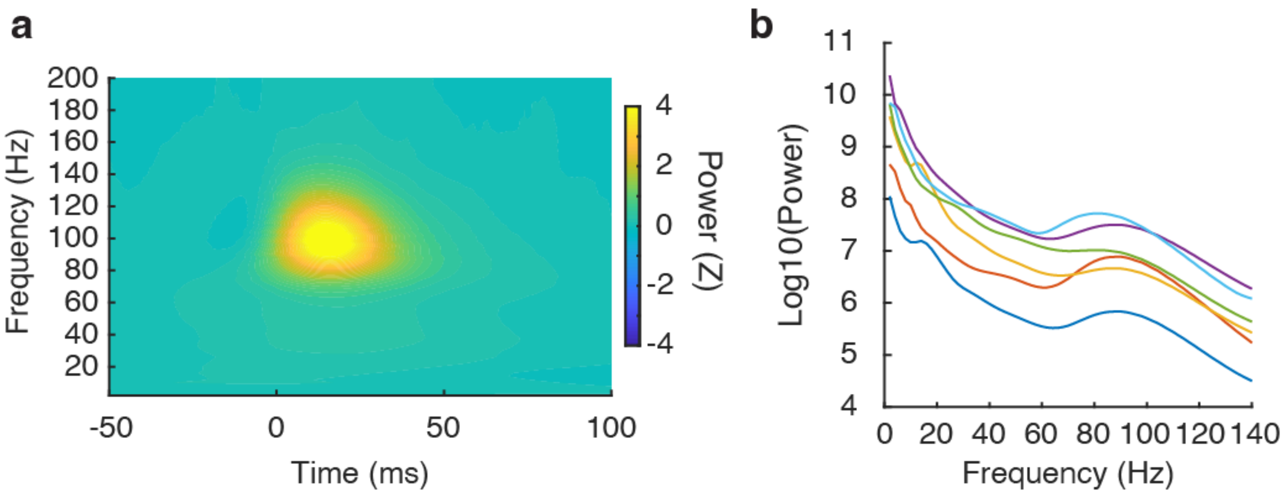

Spectral characteristics of ripple oscillations. a) Average time-frequency plot triggered to MTL ripples for the macro-iEEG contacts under consideration for our analyses. t = 0 denotes the onset of the MTL ripple. b) Raw wavelet power spectral density for detected ripple events for all participants. Average peak frequency in the ripple band is 86.7 ± 1.6 Hz.
