# Supplementary material for: Backbone spiking sequence as a basis for preplay, replay, and default states in human cortex
Source: Nat Commun. 2023 Aug 7;14:4723. doi: 10.1038/s41467-023-40440-5 (PMC10406814; doi:10.1038/s41467-023-40440-5)
Supplement: Supplementary file 2 — Reporting Summary [file 41467_2023_40440_MOESM2_ESM.pdf]

nature portfolio

Corresponding author(s): Alex Vaz, MD, PhD
Kareem Zaghloul, MD, PhD

Last updated by author(s): Jun 6, 2023

Reporting Summary

Nature Portfolio wishes to improve the reproducibility of the work that we publish. This form provides structure for consistency and transparency in reporting. For further information on Nature Portfolio policies, see our Editorial Policies and the Editorial Policy Checklist.

Statistics

For all statistical analyses, confirm that the following items are present in the figure legend, table legend, main text, or Methods section.

n/a	Confirmed	
☐	☒	The exact sample size (*n*) for each experimental group/condition, given as a discrete number and unit of measurement
☐	☒	A statement on whether measurements were taken from distinct samples or whether the same sample was measured repeatedly
☐	☒	The statistical test(s) used AND whether they are one- or two-sided<br>*Only common tests should be described solely by name; describe more complex techniques in the Methods section.*
☐	☒	A description of all covariates tested
☐	☒	A description of any assumptions or corrections, such as tests of normality and adjustment for multiple comparisons
☐	☒	A full description of the statistical parameters including central tendency (e.g. means) or other basic estimates (e.g. regression coefficient) AND variation (e.g. standard deviation) or associated estimates of uncertainty (e.g. confidence intervals)
☐	☒	For null hypothesis testing, the test statistic (e.g. *F*, *t*, *r*) with confidence intervals, effect sizes, degrees of freedom and *P* value noted<br>*Give P values as exact values whenever suitable.*
☒	☐	For Bayesian analysis, information on the choice of priors and Markov chain Monte Carlo settings
☒	☐	For hierarchical and complex designs, identification of the appropriate level for tests and full reporting of outcomes
☐	☒	Estimates of effect sizes (e.g. Cohen's *d*, Pearson's *r*), indicating how they were calculated

Our web collection on statistics for biologists contains articles on many of the points above.

Software and code

Policy information about availability of computer code

Data collection	iEEG data were acquired using Nihon Khoden's EEG data acquisition software (NeuroWorkbench version 7-02). Single unit data were acquired using Blackrock Microsystem's NeuroPort Central Suite (v 7.0.3.0).
Data analysis	Imaging data were analyzed using both FSL Brain Extraction Tool (BET), FLIRT software packages, and a Talairach daemon for the N27 standard brain. We also used the OsiriX Imaging Software DICOM viewer package. Single units were isolated using Plexon Offline Sorter. All visualization and statistical analyses were performed using custom code in Matlab, R20a (Mathworks, Inc.).

For manuscripts utilizing custom algorithms or software that are central to the research but not yet described in published literature, software must be made available to editors and reviewers. We strongly encourage code deposition in a community repository (e.g. GitHub). See the Nature Portfolio guidelines for submitting code & software for further information.

Data

Policy information about availability of data

All manuscripts must include a data availability statement. This statement should provide the following information, where applicable:

- Accession codes, unique identifiers, or web links for publicly available datasets
- A description of any restrictions on data availability
- For clinical datasets or third party data, please ensure that the statement adheres to our policy

The data that support the findings of this study are available for public download at https://research.ninds.nih.gov/zaghloul-lab/downloads

Human research participants

Policy information about studies involving human research participants and Sex and Gender in Research.

Reporting on sex and gender	Sex and gender were not considered in the study design as patient recruitment was purely driven by clinical need. Sex and gender were assigned based on participant self-reporting. We did not perform a sex or gender based analysis due to the uniqueness and small sample size of the data (N = 6 participants), but we believe that the results of our study are largely applicable regardless of sex or gender.
Population characteristics	We collected single unit spiking activity from the anterior temporal lobe in in 6 participants diagnosed with medically intractable epilepsy (2 female; 34.8 ± 4.7 years; mean ± SEM).
Recruitment	Participants were recruited on the basis of clinical need for surgical epilepsy localization. Patients were consented for both surgical management of epilepsy and participation in research studies. There were no patients that were selected or excluded based on patient characteristics.
Ethics oversight	National Institutes of Health Institutional Review Board (IRB)

Note that full information on the approval of the study protocol must also be provided in the manuscript.

Field-specific reporting

Please select the one below that is the best fit for your research. If you are not sure, read the appropriate sections before making your selection.

[x] Life sciences   [ ] Behavioural & social sciences   [ ] Ecological, evolutionary & environmental sciences

For a reference copy of the document with all sections, see nature.com/documents/nr-reporting-summary-flat.pdf

Life sciences study design

All studies must disclose on these points even when the disclosure is negative.

Sample size	Sample sizes were chosen purely in light of clinical considerations. Every participant will have a different arrangement of implanted surface and depth electrodes based on their respective seizure characteristics. Given this variability and based on our previous work (Vaz et al., Science, 2020), no explicit power calculations were performed to determine sample sizes.
Data exclusions	We excluded any channel or trial that exhibited significant noise (described in Methods). These parameters are additionally specified in the original data set, collected in Vaz et al., Science, 2020.
Replication	We did not replicate these results in a separate cohort of participants. The data presented here were captured over three years from intracranial recordings in human neurosurgical patients receiving treatment for epilepsy, and are thus extremely rare.
Randomization	Randomization of participants was not relevant to this study and participants were therefore not allocated into separate groups. Every participant completed an experimental session in which analyses were conducted comparing neural activity between task conditions.
Blinding	Blinding was not relevant to this study as there was no group allocation during data collection or analysis. All participants performed the same behavioral task, and data were analyzed for each participant who completed at least one full experimental session.

Reporting for specific materials, systems and methods

We require information from authors about some types of materials, experimental systems and methods used in many studies. Here, indicate whether each material, system or method listed is relevant to your study. If you are not sure if a list item applies to your research, read the appropriate section before selecting a response.

Materials & experimental systems

n/a	Involved in the study	
[x]	[ ]	Antibodies
[x]	[ ]	Eukaryotic cell lines
[x]	[ ]	Palaeontology and archaeology
[x]	[ ]	Animals and other organisms
[x]	[ ]	Clinical data
[x]	[ ]	Dual use research of concern

Methods

n/a	Involved in the study	
[x]	[ ]	ChIP-seq
[x]	[ ]	Flow cytometry
[x]	[ ]	MRI-based neuroimaging
